# Clinical Holistic Medicine: Classic Art of Healingor the Therapeutic Touch

**DOI:** 10.1100/tsw.2004.11

**Published:** 2004-03-04

**Authors:** Søren Ventegodt, Mohammed Morad, Joav Merrick

**Affiliations:** ^1^ The Quality of Life Research Center, Teglgårdstræde 4-8, DK-1452 Copenhagen K, Denmark; ^2^ Division of Community Health, Ben Gurion University, Beer-Sheva, Israel; ^3^ National Institute of Child Health and Human Development, Office of the Medical Director, Division for Mental Retardation, Ministry of Social Affairs, Jerusalem and Zusman Child Development Center, Division of Pediatrics and Community Health, Ben Gurion University, Beer-Sheva, Israel

**Keywords:** quality of life, QOL, human development, holistic medicine, public health, manual medicine, therapeutic touch, Denmark, Israel

## Abstract

Touching is often a forgotten part of medicine. The manual medicine or therapeutic touch (TT) is much more powerful than many modern, biomedically oriented physicians think. Pain and discomfort can be alleviated just by touching the sick area and in this way help the patient to be in better contact with the tissue and organs of their body. Lack of presence in the body seems to be connected with many symptoms that can be readily reversed simply by sensitive touch. When touch is combined with therapeutic work on mind and feelings, holistic healing seems to be facilitated and many problems can be solved in a direct and easy way in the clinic without drugs. This paper gives examples of the strength of manual medicine or therapeutic touch in its most simple form, and points to the power of physical contact between physician and his patient in the context of the theory and practice of holistic healing. Intimacy seems highly beneficial for the process of healing and it is very important to distinguish clearly between intimacy and sexuality for the physician and his patent to be able to give and receive touch without fear and without holding back emotionally.

## INTRODUCTION

The emergency room is the place in the hospital where physicians can be rewarded with immediate gratification as they treat and repair people who come with acute damage to the arms, legs, stomach, back, eyes, or ears. It is quite amazing how far you can get towards improvement and relief of problems just with a handful of simple steps and a thorough understanding of the human body. In brief, it is possible to divide injuries into visible or external injuries that can be taken at face value and internal, less visible injuries that require the assessment of a skilled physician and sometimes also X-ray, ultrasound, CT scanning, and blood tests. A truly experienced physician should be able to see at a glance whether a patient is acutely or chronically ill, whether the patient is in imminent danger, as might be the case following a stroke or a heart attack, or a perforated appendix.

Here the principles are the good intention to help, combined with often very simple steps and procedures — some words of comfort, assuring the patient that it will grow together again, heal nicely, and become as good as new; a bandage; perhaps a splint or plaster; and perhaps a painkiller or two. While there are new tools such as CT scanning, which help in making a better diagnosis and replace the guesswork and clinical assessments of former times, most treatment options are the classical ones.

The modern physician does not seem to believe in the healing power of the therapeutic touch (TT), but the simple manual procedures are not all that the skilled and holistically oriented physician can do with his hands. Since ancient times, all the way back through history to Hippocrates (460–400 BCE), the physician has used his hands to cure[1]. Many of these treatments are not used today and are looked upon as inefficient, meaningless, or even, when it comes to the famous pelvic massage of Hippocrates (a cure for "hysteria" or in-balance in the sexual energies in use up to 1900), a nonmedical sexual stimulation, practically condemned as sexually abusive[2].

There seems to be a general fear of intimacy in the medical community of our time because intimacy is taken to be the first step towards sexuality. But it is very important to distinguish between intimacy and sexuality and to distinguish between the physical contact between physician and patient in the intention of healing and sexual behavior in the intent of pleasure. Intimacy is a prerequisite for holistic healing, while physicians ever since Hippocrates have agreed that sexual behavior between a physician and his patient is unethical, destructive for the integrity of the patient, and for the professional relationship and therefore forbidden.

## THE HOLISTIC PROCESS OF HEALING

New understanding of the holistic process of healing seems to cast new light on these old procedures, which could give them a renaissance in the medical clinic. The life mission theory[3,4,5,6,7,8] states that everybody has a purpose of life and happiness comes from living out this purpose and succeeding in expressing the core talent in life. To do this, it is important to develop as a person into what is known as the natural condition, a condition where the person know himself and uses all his efforts to achieve what is most important for him. The holistic process theory of healing[9,10,11,12] and the related quality of life theories[13,14,15] state that the return to the natural state of being is possible whenever the person gets the resources needed for existential healing. The resources needed are, according to the theory, awareness, respect, care, acknowledgment, and acceptance with support and processing in feeling, understanding, and letting go of negative attitudes and beliefs. The preconditions for holistic healing to take place are trust and the intention that the healing *will* take place. Existential healing is not a local healing of any tissue, but a healing of the wholeness of the person, making him much more resourceful, loving, and knowledgeable of himself and his own needs and wishes. In letting go of negative attitudes and beliefs, the person returns to a more responsible existential position and an improved quality of life. The philosophical change of the person's healing is often a change towards preferring difficult problems and challenges, instead of avoiding difficulties in life[16,17,18,19,20,21,22,23]. The person who becomes happier and more resourceful often also becomes more healthy, more talented, and more able to function[24,25,26].

In this paper, we will acknowledge the fine art of simple, local, and therefore mostly nonholistic manual medicine or therapeutic touch with a number of examples of complete healing with simple mechanical means. This is the kind of procedure by the physician that most people acknowledge and appreciate. In a series of planned papers on clinical holistic medicine, we will show how manual medicine or therapeutic touch, combined with work on mind and feelings, can give healing to the patient on a more global and profound level in accordance with the holistic process theory of healing and the related theories.

## PHYSICAL INJURIES

The body is frail. The flesh is easily injured. Many structures such as the skin, muscles, cornea, eardrum, and mucous membranes are vulnerable. If we regard the body as a mechanical structure or machine, there is every reason to worry. It practically is designed to break by normal use. Obviously, life can only take such liberties with the design because the body has such tremendous healing potential. If something breaks, it will knit together again. This means that a great deal of the treatment is explaining to the patient that there is no cause for worry, because the good body will take care of itself if you leave it to heal in peace. Sometimes, though, we can do much to help promote the healing process. Below are three short case reports (as taken from the notes of the family physician) that describe classical medical treatment of physical injuries:

> **Male, aged 36 years with an eye injury:** The patient was cutting stone with an angle grinder without wearing glasses and received a glowing metal chip (1 mm × 0.3 mm) in the left cornea at 7 o'clock, outside the central vision. Chip removed with a needle point without any visible epithelial damage following anesthesia. Treatment: eye pad and eye drops. He was instructed to return in 3 days if pain persisted.

The cornea of the eye consists of an external mucous membrane, the epithelium, under which is a crystalline layer of protein fibers that turn opaque when exposed to heat because of coagulation (as with egg white). However, the greatest damage to organs is often caused by the body seeking to heal them. If the foreign object is removed, the patient can avoid a lengthy and painful inflammatory healing process, which in the worst case may cause permanent damage to the cornea. The art is to remove the foreign object without causing damage. When that is not possible, the physician must avoid causing more damage than the natural healing process would do. In this case, the chip that was burnt into the cornea of a 36-year-old worker was removed without any significant damage. The patient was more frightened than hurt.

> **Male, aged 45 years with a burst eardrum:** The patient burst an eardrum when blowing his nose yesterday. Examination: small cut, 1/4 of the eardrum diameter, neatly closed with coagulum. Should return to consultation in 3 weeks, if hearing is not back to normal.

It is the most exalted job of the physician to convince the patient that the body is strong enough and will heal without any problems. Naturally, the latter is not always true, but we believe that the lack of confidence may delay the healing of damaged tissue if it leads to a withdrawal of attention from the area. The bad habits that may spring from the patient's lack of confidence in the body following a trauma can also lead to more damage. If you do not trust your foot to carry you after an injury, protracted incorrect use of and putting of overload on the foot may lead to new injuries.

> **Male, aged 45 years with scar tissue:** Keloid formation — hard scar tissue 40 × 3 × 3 mm above right heel — corresponding to wound caused earlier by the hoof of a horse. Gentle massage. Prognosis: will pass in the course of 6 months. The patient can continue to do his own massage.

Wounds may heal with considerable keloid formation. We believe that recovery will be quicker if we massage the tension and shock out of the wound, but there is no scientific evidence of this. Under any circumstances, the patient will become familiar with the scar during the massage and will often feel the problem diminishing, whether the keloid disappears or not.

## PAIN IN THE LOCOMOTOR SYSTEM

Pain in the locomotor system constitutes the greatest complaint by people in the western hemisphere, although it is actually quite easy to relieve. Nevertheless, many adults and children do not get the help they need from their family physician or general practitioner (GP) and therefore have to live with the pain through large parts of their lives. In any schoolyard, you can find children sitting on benches with growing pains, unless they are limping about playing. Many cases of stomachache in children are caused by tension in the abdominal muscles because of fear or insecurity. It is often easy to remedy these problems if the GP and the parents are willing to consider what lies behind them.

> **Male child, aged 13 years with knee pain and growing pain:** Pain in left lower leg 3 cm below the knee and laterally. Examination: there is no pathology around knee or leg. Only finding is a very tender area 3 × 3 cm about a handsbreadth below the knee, which is massaged until free from pain, when walking no longer hurts. Instruction in self-treatment.

A young boy comes limping into the surgery. His knee hurts and he can barely walk. After 10 min, the painful area has been identified and massaged until free from pain. Miraculously, the boy has a normal gait again without any complaints. Contact, touch, reassurance, confrontation of the pain, and when the patient becomes present in the knee again, the symptoms are gone. We wonder whether it is mostly in the boy's consciousness that the status of the knee is upgraded from bad to healthy?

Pain that vanishes on touch can be instructed and explained to the mother in order to touch her child, be attentive, and the pain will often disappear. We work with holistic medicine and because we approach the cause of the problem, we are able to help the boy.

> **Female, aged 36 years with knee pain after fall:** She fell on her knee 3 months ago with no swelling or discoloration at the time. Her knee is still hurting. She believes that there is a bone fragment or similar. Pain localized in subcutis just below a scar on the knee that becomes visible with the leg extended. Knee is massaged almost until free from pain. The patient is encouraged to let partner massage 10 min each day for 2 weeks. Should return if the problem persists.

Children are not alone in finding it difficult to repossess their bodies after a trauma. Often people really truly hurt, day in and day out, until someone asks: “Is *this* where you hurt?” pressing the fingers right into the pain and making the patient catch his breath and then breathe it all out, letting go of whatever he is unconsciously guarding, the painful site. *Letting go* is a simple cure, as old as the art of healing, but truly difficult for people of the world today, who keep hanging on to it all.

> **Female, aged 28 years with sprained ankle:** Industrial injury yesterday — stumbled and twisted her ankle. Slight swelling around left ankle laterally, claims having difficulties making her toes “work”. Examination: no indirect tenderness, direct tenderness corresponding to strokes from lateral epicondyle to forefoot. No loss of sensitivity. Protective immobilization of sprain. Instruction: able to work. Prescribe mobilization until pain threshold, analgesics if required. Estimated time before healing 2–3 weeks.

Some of the joys of being a physician come from knowing the natural course of healing. Once you pass this knowledge on to the patient, who then accepts the situation, the problem is often solved. Pain or discomfort for 3 weeks is quite acceptable once you know that this is how it is supposed to be. So a brief talk to make the patient aware of this is very valuable.

> **Male, aged 21 years with back problems after football:** This patient has had back pain for 2 months, since he fell on a hard surface, landing straight on his back while playing football. Examination: spine: normal tension corresponding to neck and shoulders, upper and lower back. It is possible to induce the familiar pain by pressing on the tense muscles, but not by pressing the spinous processes. No doorbell syndrome, no subluxations. Recommended exercises: press-ups, bending forward as in touching the toes. Break the vicious circle by relaxing and exhaling when it hurts instead of getting tense. Should return if the problem persists.

Many people see the back as a weak structure. It is not, quite the contrary. When people get "back trouble”, it is mostly mild sprains or minor muscular problems that will normally resolve easily with training and exercises. Every now and then a patient does not willingly accept the thesis that the back is strong. When some people refuse to drop the idea that “their back is weak”, until they have had psychotherapy, it is because of the important symbolic meaning of the spine. A patient who subconsciously feels “spineless” cannot have a strong back.

There is a lot of rough play on the sports fields and young men may suffer some serious tissue damage when playing football or doing other sports. Fortunately, the body has great healing potential, so all they really need to worry about is the head (brain) and knees (cruciate ligaments). The knee seems to be the football player's weakest link. Remaining aware of the knee during the game is the best form of prevention, and it can be developed through different exercises in enhanced presence in the body.

> **Female, aged 24 years with back pain:** Back pain. T2–T12 very tense and tender, almost arched, manipulated into place with 10–20 loud snaps following a brief talk about why the patient is tense: she is moving and is finding it very problematic. Afterwards she feels much better.

Back pain is another widespread complaint often encountered in medical practice. Often people do not realize or simply ignore the correlation between major acute problems in life and stresses in the back. As soon as they become aware of the correlation, they virtually hit jackpot. The back pain is almost gone in a minute and the problems resume their full emotional scope. It seems possible to temporarily “park” acute emotional problems as stresses in the back. Obviously this does not solve the problems, but rather makes them worse.

> **Male, aged 74 years with “frozen shoulder”:** After helping out during a removal, the patient had pain in the left arm and he found it difficult to lie on the arm or carry things. There is a slight tingling down into the fingers. He has trouble getting dressed. Examination: distinct tenderness corresponding to lateral end of left supraspinatus muscle and tenderness in the shoulder. No lowered sensitivity, but reduced strength in brachial muscles, probably because of protective reaction. Diagnosis: tendinitis in left supraspinatus muscle without rupture. Intervention: referral to physiotherapy and prescription for analgesics and to avoid lifting heavy objects with arm for 2–3 months, but remember to use it.

Protective immobilization can be deceptive. There is nothing wrong except pain or tenderness, but the whole arm may have lost almost all its strength. A few carefully chosen remarks to the effect that all is well will often, as in this case, restore a lot of the strength. And in our opinion, the arm should be used with care and not spared completely. It should be exercised and used to the pain threshold.

## INFECTIONS

The human body would be eaten by viruses, bacteria, fungi, and other parasites within just 24 h if not for an effective immune defense system. At the microbial level, we fight bravely for our survival. Once the bacteria get the upper hand for a while, the alarm is sounded in the immune system. Only then do we become aware of their existence. Infections are some of the most frequent disorders in general practice. Infections come about when the immune system is weak, which is often the case when a person is not thriving.

> **Male, aged 41 years with a chronic throat infection:** Severe throat complaints with pain and recurrent tonsillitis. Examination: oral cavity: larynx swollen and tender, no coating. Strep A negative; distinct pain corresponding to hyoid bone, which is unlikely to be related to the inflammation; three large lymph nodes bilaterally along the sternoclaidomastoideus muscle. Diagnosis: chronic throat infection. We talk about potential causes of this problem and it turns out that the patient sings and has to force his voice. It began with a throat infection. Pain probably due to tension in the throat because of incorrect use. He needs to work with his voice and learn to relax. We shall wait and see.

A chronic inflammation of the throat is interesting in that it often appears to be completely or almost sterile. It is not caused by bacteria, but rather by an internal disturbance in the body itself that the patient can suppress with training, exercises, and working with his or her consciousness, while seeking the cause of the disease.

## EDEMA AND ALLERGY

Several other conditions can be relieved by some simple steps and/or patience. Let us look at some of them in the next case records.

> **Female, aged 20 years with ankle edema (swelling):** Edema in lower limb, stands a lot. Auscultation of the heart: normal. Diagnosis: suspected idiopathic [not caused by another disease] ankle edema. Prescribe exercises to activate skeletal calf muscle pump. Should come back for follow-up to talk about body use, when standing at work.

A young woman should not suffer swollen legs. There is some imbalance that she ought to get rid of. But how deep can you go when talking to a young woman living a fast life? So we provide a "quick fix" and tell her that she can come back for more. She does not feel the need to off-hand. She is given ankle-flexing exercises, but not diuretic drugs.

> **Female, aged 51 years with an itching eye:** Allergic conjunctivitis [a sterile inflammation of the conjunctivae, the mucous membranes covering the eye] in right eye, intensified by her scratching. We try prescribing eye compress and eyedrops.

Another classic: When it itches, you scratch, making the itching worse, and then you scratch even more. Where does it end? In this case the patient had to have her eye covered. That broke the circle and the eye was allowed to heal.

> **Male, aged 35 years with pain in the scrotum:** Presented with very tender right side of scrotum a week ago, now no complaints except for worry. He was informed that the

> finding is quite normal, and so is varicocele [swelling of the scrotum]. Should return in the event of acute problems.

Embarrassing parts of the body, the parts most often the objects of teasing when we are little, are hard to relate to and often problematic.

## PSYCHOLOGICAL AND SOCIAL PROBLEMS

Often a patient consults a physician about problems that are not physical. Our mentality and our environment are intricately connected, so here we are really put to the test. To help a patient with psychological problems, he or she will often need to work on perception of life and human nature. Many times it does not take a lot to achieve great results. The key to the matter is to put the patient on the right track and the rest will follow. In the case of children, often the parents are behind the problem and the ones in need of help. Here also physical touch and holding seems mandatory for success. Often this is not even mentioned in the case record as it is taken for granted. Sometimes the holding is solely emotional, but of the same intense quality as physical holding. This is “holding without holding”, a beautiful art that can solve many problems.

> **Girl, aged 5 weeks with crying:** 5-week examination. She develops normally and well. Parents believe she has colic. She is very hot because her parents dress her too warmly for the weather, 25–30ºC. She stops crying when they take some of her clothes off and begins again when they put her clothes back on. Guidance.

Parents who cannot make their child stop crying will often say that it has “abdominal colic”, spontaneous stomachache of unknown cause. In our experience, it is always possible to make the child stop crying if you can satisfy its needs (perhaps except for children with severe brain damage). This child was undressed in the summer heat and immediately stopped crying. After the examination, the parents dressed their child again, who immediately started crying. It is sometimes a mystery how parents can be so unobservant. It gives a feeling that really skilled specialists are needed in childcare. Modern physicians do not believe that “colic” can explain why children cry, but in the old days this was considered a reasonable explanation. We have a patient who was left to cry in the stairway for 3 months on end. Obviously it is not acceptable to neglect and maltreat infants in this manner.

> **Girl, aged 3 years with a fussy mother:** The girl is thriving and looking well, but mother thinks she has a sore throat. Examination: tonsils swollen, but not red or coated. General health not affected. Diagnosis: viral infection. Wait and see for 8 d.

The “fussy mother” torments people around her. It is difficult to inspire a mother with confidence that her girl is strong and able to cope if she fundamentally lacks faith in life. We simply ask the girl, who says that she is fine. If she is not the least affected she has the last word on the matter.

> **Female, aged 25 years and a single mother:** (1) Ear pain that turns out to stem from a major blockage/muscle tension in the neck, which is massaged. (2) Crying. She has many problems with her son, hard to be a lone parent. Cannot manage the son who is far too active and domineering. She wants to do really well for him. We talk about her defining her own limits instead of setting limits for her son, which could be the cause for the tension in the neck. Instructed not to hit the child. Invited to come back to talk problems over.

Things often tie together and often the whole situation crystallizes as soon as the patient receives help in analyzing it. Here, the mother is having trouble controlling herself, which is taken out on the child, whom she feels she cannot manage. She is tense and hurts, which makes her torment her child even more. With enhanced consciousness and acknowledgment things slowly fall into place.

> **Female, aged 61 years with severe overweight and cervical problems:** Weight 115 kg. Present with problems with her neck, which locks in an anteflexed position [bending forward]. Has seen a chiropractor who said there is no physical obstacle to her lifting her head. Examination: lumbar lordotic curve much enlarged and compensated by a much enlarged kyphotic curve. No signs of spinal disorders. She is instructed to lose weight and strengthen back muscles by eating less, taking long walks, swimming 2–3 times a week, preferably doing back tension and extension exercises and exercise by "reaching down to the toes from front and side".

The holistic physician is a nightmare to any severely overweight back patient. After all, it is really your own responsibility how much you weigh and what state your back is in. We encourage them to look at themselves from the outside, assume responsibility, and do something about their situation. Either we get through to them or they look for another doctor with a more “understanding” attitude. In our opinion the latter option would be likely to lead to a long career as a patient in the healthcare system.

> **Female, aged 38 years with overweight:** Slimness plan. 131 kg. Presents fully motivated and has made the following plan: (1) swimming twice a week, (2) cycling to town and to bingo, (3) eating low-fat food, mostly crisp bread with low-fat cheese. We talk about how diet should include many vegetables and little meat. She intends to live off her fat deposits and wants to lose 31 kg over the next 31 weeks to weigh 100 kg. This means lowering her daily calorie intake to two-thirds of current level. We talk about how to mentally overcome the sensation of hunger. Check-up every month.

A patient who has made her own plan for slimness should mean business. For some reason, it is almost as difficult to stop overeating as it is to quit smoking. We believe in addressing the dependency directly, but this is often very tough on the patient. Beneath a pattern of eating disorders the patient often has some serious existential problems.

## SEXUAL PROBLEMS AND PAIN DURING INTERCOURSE

Discomfort during intercourse is more likely to have a psychological than a physiological explanation. A large amount of superfluous testing is done because people are not in sufficient contact with their private parts[27].

> **Female, aged 35 years with lack of resources:** For the past 4 weeks, this patient has suffered from pain, dizziness, headache, and fatigue with perhaps a slightly elevated temperature. Pain during intercourse. Two young children, full-time job, marital problems. Examination: a patient who is tired with few places to refuel. Pelvic examination: tenderness in upper left side of the vagina. Sterilized, faithful to her husband. Inspection: vulva, vagina, vaginal orifice all natural except from some yellowish discharge (microscopy): an imbalance due to absence of lactobacilli [lactic acid bacteria], otherwise normal, no clue cells [cells covered by tiny bacteria only visible under a microscope]. Exploration: no adnexal tenderness or cervical motion tenderness corresponding to the uterus, otherwise as stated above. Auscultation of the lungs and heart: normal. No focus found. Diagnosis: limited resources. Prescribe lactic acid bacteria vagitories.

When the energy of life is low, the patient often develops symptoms from all organ systems — from headache to abdominal complaints. This patient feels that her body is “slipping”, but she does not recognize the pattern behind it, namely that she lives all wrong and that is what her symptoms are about. It is easy to see from the outside. Although we owe it to the patient to examine her thoroughly, the conclusion is almost given beforehand Two young children and a full-time job is a lot; marital problems into the bargain clearly too much. She should stay home from work for a week and reconsider her life. Her body tells her that she has reached the limit. But will she understand? And is she ready to do something about it?

The classic art of healing is still a great part of the work of a GP and in daily practice we often call it “manual medicine”. It is based on a solid understanding of the human body, the mind, and the correlation between them causing the symptoms that are bothering the patient. This understanding will help the physician to work on the relevant attitudes and behavior of the patient, so that the patient becomes present in the body and uses the body in a better manner. The body is relieved of some of the pressure and the self-healing forces are set free. As illustrated by the above cases, it often takes surprisingly little and the effect may well be substantial and lasting.

In manual medicine, we work with the body as it is. This imposes some limits on how far we can go; limits that can be expanded considerably by the experienced physician.

## DISCUSSION

There are many ways of touching, like therapeutic massage (TM), healing touch (HT), Reiki or therapeutic touch (TT), which can be classified as energy or metaphysical therapies, one of the five domains created by the National Institutes of Health (NIH) Office of Complementary and Alternative Medicine (CAM)[28]. A Medline (PubMed) search on January 29th, 2004 found 470 records under the heading of TT. Many studies are published in nursing journals with evidence that TT has the ability to cause relaxation, also laboratory evidence for physiological change towards relaxation and improved immune function, but the last entry was a study on improvement of symptoms in cancer[29]. This was a randomized, prospective, two-period, crossover intervention study[29], where the authors tested the effects of TM and HT in comparison to presence alone or standard care, in inducing relaxation and reducing symptoms in 230 subjects. TM and HT lowered blood pressure, respiratory rate, and heart rate. TM lowered anxiety and HT lowered fatigue, and both lowered total mood disturbance. Pain ratings were lower after TM and HT, with a 4-week treatment with nonsteroidal and anti-inflammatory drug useless during TM. There were no effects on nausea. Presence reduced respiratory rate and heart rate, but did not differ from standard care on any measure of pain, nausea, mood states, anxiety, or fatigue. This treatment was an addition to receiving cancer chemotherapy.

We see problems mainly connected to three areas: pain and damage, love and intimacy, gender and sexuality with ethical and legal problems included.

### Pain and Damage

Often, when we work with physical pain in the clinic, like knee pain, we must actually enhance the pain to make the patient confront it and integrate this area of the body with the connected chock (trauma with overwhelming pain) and feelings. It is extremely important to discriminate between pain that feels like something is wrong or being destructed (*destructive pain*, as when some bone is broken inside, and you create a pressure on the wounded periost) and therapeutic pain (called *paradoxal pain*) as when sore shoulders are massaged, which hurt in a good, comforting way. Intense paradoxal pain is often needed to heal strong physical locomotor pain[30], and it is very important that the patient fully understand the integrative process. If not, you can be accused of causing a worse condition than before and you now become the cause of the condition in the mind of the patient. This is a self-defense on the part of the patient, not wanting to assume responsibility for his/her own pain and not wanting to go into this area of the body and the connected often strong and negative feelings from a personal past. So the physician's whole attitude towards pain is of extreme importance for the success of the therapeutic touch. The physician must be loving and holding on one side and firm (in a way merciless), direct, and determined and totally without pity for the patient on the other. To accomplish this, where you can hold and move the patient at the same time, is the fine art of medicine. You are to be trusted by the patient, while you inflict pain often rising to almost the same intensity as the original trauma, if just for a short moment, for the patient to confront, integrate, and heal.

There is, in contrast to the normal idea of the body's inner space as hidden, no place of the body which cannot be reached by the hands of a proficient physician, except from the areas completely cowered with bone (the brain, deep nasal cavity, spine cavity, bone marrow).

"Manipulation" is taking touch into physical movement. This is highly effective, but needs so much sensitivity for the patient's body and feelings that it is best to avoid it in many situations, not to do harm, especially when it comes to the neck. Chiropractic, high-energy manipulations of the back and neck and other joints can be very efficient in "opening" old blockages; doing this in the high neck takes a specialist or very experienced physician because of the danger of causing whiplash-like damage. The danger is not really as much that you can make a physical damage, because the force of your hands is only a small percentage of the force inflicted in a car accident, but again the patient can believe that you made further damage on his neck. Manipulation of children also takes an expert.

## Love and Intimacy

The following case record from an advanced holistic session[31] shows the intimacy needed for healing of the deeper layers of the patient's existence (love, power, and sexuality)[6].

> **Female, aged 30 years but emotionally and sexually "dead**": Eleventh conversation and sexological procedure: acceptance through touch. She is so beautiful, fine, and sensitive with a pure innocent consciousness. And at the same time, she is to a great extent completely dead. We have agreed that today I (SV) will play the role of the good father she has never had. She lies on the couch crying, and I hold her and kiss her neck and tell her that she is the apple of my eye. We talk about what enneagram subtype she is: social, sexual, or survival, and it is in her judgment as though all her problems are concerned with gender and sex. I am in agreement with this. We therefore agree to work on her acceptance of her own gender and her own sexuality through accepting touch: supportive acceptance through contact. She first holds herself on the outside of the vulva (on the outside of her briefs), with my hand supporting around hers. Afterwards I hold right on, while she holds her hand on top of mine. If she holds harder, I hold harder; if she holds more softly, I hold more softly; if she lets go, I let go too. In that way she controls the session, according to her need for support. Conversation: in the meantime, we talk about how her boyfriend only wants to do it right for her sexually, while she only wants to do it right for him. She is reading a book about women's orgasms – mostly for his sake! And when they are together, from his side it is only about her desire. It has completely gone off the rails in my opinion, but because she feels completely devoid of value, she cannot allow herself to feel any joy or desire at all. It is a Gordian knot. Here she hits the "wall" and sees far more clearly than before how ill she is and what has to be done to cure her. She sobs inconsolably, lies on her side, and asks me to hold her.

Please remark the physical and emotional closeness in the sentence: "She lies on the couch crying, and I hold her and kiss her neck and tell her that she is the apple of my eye." Touch has a completely different function here, giving the patient the holding she needs. To kiss a patient is a dangerous thing to do, as it is very important that the patient is totally clear of the physician' intention. If misunderstood, the patient could interpret the closeness as the beginning of a sexual relationship. On the other hand, is it very important that the physician does touch, when he understands that this is what is needed. The medical session is so important to the patient's future health, it is definitely not to be disturbed, slowed down, or even interrupted by negative thoughts, judgment, and lack of physical and emotional generosity. It is completely fine to take a crying patient on your lap and hold your arms around her when she is 4 years old psychologically and desperately needs that care she never had, to heal her old wounds on her soul. But always let the patient know what is going on and why, by being verbally explicit about it.

## Gender and Sexuality

Touch is a very dramatic and mutual thing for our physical and emotional being; when I touch you, you touch me[32]. As many people only know touch in connection with sexual behavior, a sexual reaction to a completely innocent touch is not rare in the clinic. If sexually aroused, the female can react as "if she has sexual organs all over her body", making physical contact with her very electric and sexual, and this reaction can come very suddenly and highly unexpected and not always convenient. If the physician retracts from her in this situation, as would be the immediate reaction of most normal people, she will experience that her body, gender, and sexuality are not acceptable, which destroys intimacy and trust for the patient and the professional relationship. Being there, staying in contact, takes a great deal of spaciousness on the part of the physician. This spaciousness should be a part of our medical training, but is often not, making so many pelvic examinations and other procedures emotionally painful and awkward for both physician and patient.

Penetration of the female vagina is normally considered to be sex, so this takes a specific medical reason and an extended contract with the patient[27,31]. Different countries have very different juridical practices, so be aware of the laws that regulate the behavior of the physician in your country.

The subject of ethics has been of utmost importance for the physician since Hippocrates and whenever the physician touches the patient, the ethics of the action must be considered. The problem of touch is mostly much more of an ethical problem than it is a legal problem: Why do you touch the patient, what is the intention? If the intention is for the physician to enjoy his patient — what we do most of the time with people in private — we consider this unethical, even if this is just holding hands. The physician should have the healing of the patient as his sole focus and if the intention of the physician is wholehearted and rooted in deep medical expertise to heal the patient (and in this intention touch any part of the body including the genital), this is ethical. Interestingly, the physician's ethics seems to be proportional with his results with his patients. Only the clearest of intentions can bring us outstanding results[22].

## CONCLUSION

Touching or therapeutic touch (TT) is an almost forgotten art of medicine and many areas of the human body cannot be touched even by the physician without a specific reason of examination or biomedical treatment.

But simply touching sensitively — the essence of manual medicine — is a much more powerful tool than many modern and biomedically oriented physicians assume. Many pains and discomforts can be alleviated just by touching the sick area and help the patient to be in better contact with the troubled tissue and organs of the body. Lack of presence in the body seems to be connected with many symptoms that can be readily reversed simply by sensitive touch in the intention of healing. When touch is combined with therapeutic work on mind and feelings, holistic healing seems to be facilitated and many problems can be solved in a direct, easy, and effective way in the clinic, without the use of drugs.

Manual medicine, even in its most simple form, is a powerful and often underestimated medical tool. The great power of physical contact between physician and patient, which is even stronger in the context of the theory, practice, and intent of holistic healing, is often not taken sufficiently into use in the medical clinic today, where everything is supposed to be cured with a drug. Much suffering and money could be saved if the physician of our time was able to discriminate more clearly between intimacy and sexuality and thus dared to be more intimate and physical with their patients. If the physician masters the art of touch he can even give the quality of holding without touching.

## ACKNOWLEDGMENTS

This study was supported by grants from IMK Almene Fond. The quality of life research was approved by the Copenhagen Scientific Ethical Committee under number (KF)V.100.2123/91.

## BIOSKETCHES

**Søren Ventegodt, MD,** is the Director of the Quality of Life Research Center in Copenhagen, Denmark. He is also responsible for a Research Clinic for Holistic Medicine in Copenhagen and is a popular speaker throughout Scandinavia. He has published numerous scientific or popular articles and a number of books on holistic medicine, quality of life, and quality of working life. His most important scientific contributions are the comprehensive SEQOL questionnaire, the very short QoL5 questionnaire, the integrated QOL theory, the holistic process theory, the life mission theory, and the Danish Quality of Life Research Survey, 1991–94 in cooperation with the University Hospital of Copenhagen and the late pediatric professor Bengt Zachau-Christiansen. E-mail: ventegodt@livskvalitet.org. Website: www.livskvalitet.org

**Mohammed Morad, MD**, is Specialist in Family Medicine, Lecturer in Family Medicine at the National Institute of Child Health and Human Development, Division of Community Health, Ben Gurion University of the Negev and the Medical Director of a large area clinic in the city of Beer-Sheva. He has publications on Bedouin health, health aspects, spiritual health, and aging in persons with intellectual disability, and is a presenter on topics such as health policy and services for the disadvantaged at national and international conferences. E-mail: morad62@barak-online.net

**Joav Merrick, MD, DMSc,** is Professor of Child Health and Human Development affiliated with the Zusman Child Development Center, Division of Pediatrics and Community Health at the Ben Gurion University, Beer-Sheva, Israel; the Medical Director of the Division for Mental Retardation, Ministry of Social Affairs, Jerusalem; and the Founder and Director of the National Institute of Child Health and Human Development. He has numerous publications in the field of child and human development, rehabilitation, intellectual disability, disability, health, welfare, abuse, advocacy, quality of life, and prevention. Dr. Merrick received the Peter Sabroe Child Award for outstanding work on behalf of Danish Children in 1985 and the International LEGO-Prize ("The Children's Nobel Prize") for an extraordinary contribution towards improvement in child welfare and well being in 1987. E-mail: jmerrick@internet-zahav.net. Website: www.nichd-israel.com
